# Computational Quantification of Mouse Retinal Vasculature Using ImageJ

**DOI:** 10.21769/BioProtoc.5705

**Published:** 2026-06-05

**Authors:** Michel Nader, Hirad A. Feridooni, Mahtab Tavasoli, Sarah Van Der Ende, Christopher R. McMaster, Johane M. Robitaille

**Affiliations:** 1 Faculty of Medicine, Dalhousie University, Halifax, NS, Canada; 2 Department of Pharmacology, Dalhousie University, Halifax, NS, Canada; 3 Department of Ophthalmology and Visual Sciences, Dalhousie University, Halifax, NS, Canada; 4 Department of Pathology, Dalhousie University, Halifax, NS, Canada; 5 Department of Pediatrics, Dalhousie University, Halifax, NS, Canada

**Keywords:** Retina, Vessels, Mice, Screening tool, Disease models, Candidate treatments, Image analysis, ImageJ, FIJI, Fractal analysis, Image processing, Morphometry

## Abstract

Postnatal mouse retinal vascular development is a widely used model for studying retinal vascular diseases and evaluating candidate therapies. This is particularly relevant for inherited disorders such as familial exudative vitreoretinopathy (FEVR), in which impaired vascular growth and organization are central to disease pathogenesis. Numerous approaches have been used to assess retinal vasculature in mouse flat mounts, ranging from qualitative descriptions to limited quantitative measurements of vascular growth. However, phenotypic variability across genetic models, including different models of FEVR, complicates comparisons and underscores the need for standardized, comprehensive multi-parameter analyses that are suitable for rapid and cost-effective screening studies. We describe a standardized morphometric protocol using ImageJ software to quantitatively analyze mouse retinal vasculature in a reproducible manner. The protocol begins with measurement of areas of vascular disorganization (meshes) as well as total vascular and retinal area. Two defined regions in the peripheral and midperipheral retina are then selected to quantify cell clusters, followed by image processing, binarization, and skeletonization. From these processed images, vascular density, branch number, branch length and thickness, junction number, triple points, and box-counting fractal dimension and lacunarity are quantified. Overall, this protocol provides a rapid, cost-effective, and standardized framework for quantifying retinal vascular phenotypes across diverse mouse models. By capturing multiple structural features and accommodating phenotypic variability, it is well-suited for comparative studies and therapeutic screening in retinal vascular disease.

Key features

• Computational method for mouse retina vessel image analysis for multi-parameter vascular quantification for user-selected regions of interest.

• Free open-source ImageJ-based workflow combining disorganization mapping, skeletonization, and fractal analysis for reproducible vascular network characterization.

• Optimized for rapid, cost-effective screening of structural vascular outcomes across developmental stages, disease states, and therapeutic interventions.

## Graphical overview


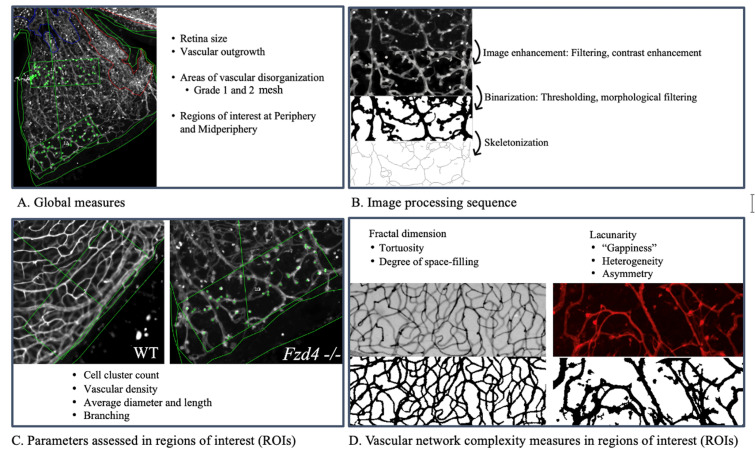


## Background

Development and maintenance of the retinal vasculature is essential for proper ocular function and vision. Disease states where retinal vascularization is abnormal are common causes of blindness [1–3] and include diabetic retinopathy [4–7], retinopathy of prematurity (ROP) [8–12], and inherited retinal vascular diseases exemplified by familial exudative vitreoretinopathy (FEVR) [9,13–29]. In humans, the retinal vasculature is initiated by vascular precursors that lay down the primary vessels that project radially from the optic disc to the retinal periphery by term gestation to form the superficial vascular plexus. From this plexus, starting around 25–26 weeks of gestation, secondary vessels penetrate the retina to form two networks, the deep capillary plexus, on either side of the inner nuclear layer [27–35].

In rodents, as in humans, a primary vascular bed first extends from the optic nerve to the peripheral retina in a single plane along the superficial retina, and the superficial plexus then sends sprouts vertically into the deeper layers of the retina that will form two intraretinal networks. Unlike humans, who exhibit fully formed retinal vessels by term birth, the primary vascular plexus in rodents develops in the first postnatal week, and the formation of the deeper layers is completed by postnatal day 14 (P14). Rodent models offer a unique opportunity to study the evolution of developmental retinal vascular disorders [29,34,36] and, as such, are widely used to study mechanisms of human retinal disease. To do so, retinal vasculature is often determined by flat mounts of retinas followed by staining, visualization, and analysis. However, there is no standard approach that can quantify relevant parameters and capture the phenotypic diversity observed in the various genetic and environmental mouse models, comparing them and assessing responses to candidate treatments. An extensive number of parameters can be individually quantified (Tables 1 and 2). We devised and tested a computational protocol for determining clinically relevant retinal vascular properties ranging from size to network complexity.

Other computational tools have been published in the past, such as Angiogenesis Analyzer, REAVER, Vessel Analysis, AngioQuant, RAVE, SIVA, and AngioTool, among others [37–43]. However, they do not have a wealth of scientific literature regarding their application, they are inflexible regarding which parameters are quantified and how, they are not customizable, they do not have an active developer community, most are not open source, which prevents full transparency regarding the underlying algorithms, they present financial barriers, and they are not available on multiple operating systems. Our protocol thus responds to all of these limitations by using ImageJ, a free and fully open-source software with an active community, vast customizability, compatibility across iOS, Windows, and Linux operating systems, and extensive use in the literature [44].

Global vascular development of the mouse retina depends on the postnatal age and body weight and is measured as percent vascularized area (PVA) of the total retinal area and vessel density (VD). VD is affected by the number, length, and diameter of the vessels. Therefore, we include average branch length (BL), average branch thickness (BT), number of branches (BN), number of junctions (JN, a point where a branch divides), and triple points (TP, junctions where there are two branches emerging from one) in regions of interest (ROIs) (Table 1). Together, these parameters can clarify whether a large VD consists of many small, interconnected capillaries or fewer but larger vessels.

**Table 1.** Parameters and techniques used to quantify retinal vascular networks and their relevant regions of interest (ROIs)

Parameter	Description	Reference
Retinal area	Total area of the whole retina	N/A
Percent vascularized area	Proportion of total retinal area with vessels	N/A
Vascular outgrowth	Distance from optic nerve to vascular front	[36]
Vessel density	Area of the vessels divided by the total area of the ROI	[36,38,45]
Histogram integrated density	The product of the length of the fibers and the average radius	[46,47]
Vascular length	Total length of skeletonized vessels	[36,38,45–48]
Average branch length	Average length of all the branches in the network	[48–50]
Branch length density	Average branch length per area of ROI	N/A
Vessel length density	Total vessel length per area of ROI	[38,45–47]
Characteristic length	Total length of all centerlines (skeleton) divided by the number of intersections	[45–47]
Average vessel diameter	Average vessel diameter/thickness	[45–47]
Vessel diameter density	Average vessel diameter per area of ROI	N/A
Diameter histogram	Histogram of measured diameters with mean, mode, median, standard deviation, skewness, and kurtosis	[46,47]
Vessel Perimeter Index	Ratio between vessel perimeter and total area	[49–51]
Branchpoint density	Number of branching points in the skeletonized network per area	[36,38,45]
Branching angle	The first angle subtended between two daughter vessels at each branch point	[50–52]
Branching coefficient	Coefficient relating the diameter of the parent branch and the two daughter branches according to an equation	[51–53]
Vessel segment partitioning	Number of segments divided by total vessel segment lengths	[52–54]
Nearest neighbor distance	Average minimum distance and variability in distribution between the vessels in the region	[36,38,45]
Tortuosity	Measure of the degree of twisting and turning in the path of the vessel	[51–53]
Vessel Complexity Index	Index relating vessel perimeter to area	[51–53]

Parameters such as VD, BL, BT, BN, JN, and TP rely on the assumption that defined vessels are present in a ROI, but this is not always the case. Retinas from a genetic mouse model of the childhood blinding disorder FEVR, the *Fzd4-/-* mouse model, may manifest large areas of vascular disorganization—termed *meshes*—with no defined vascular network. To capture these characteristics, each of these areas is outlined and measured, and its ratio to the total vascularized area is reported. We define meshes with a significant lack of defined vasculature as Grade 1 mesh, and areas with an almost complete lack of structure as Grade 2 mesh.

**Table 2.** Examples of fractal analysis methods

Name	Reference
Box-counting fractal dimension (D_B_)	[53–55]
Information dimension	[53–56]
Correlation dimension	[53–56]
Local connected fractal dimension	[56–58]
Differential box-counting method	[59–72]
Extended box-counting method	[61–63]
Isarithm method	[62–64]
Blanket method	[64–66]
Triangular prism method	[65–67]
Hausdorff fractal dimension	[67–69]
Modified Hausdorff fractal dimension	[67–69]
Fourier fractal dimension	[68–71]

The term complexity can relate to branching, vessel overlap, tortuosity, density, symmetry, space-filling, and the heterogeneity of any of these factors. Quantifying this complexity in our vessel networks can be addressed by applying fractal geometry (Table 2). In combination with the other quantified structural characteristics of this vessel network (e.g., length, thickness, and number), we can glean information on both the development of vessels at the local level and how that impacts the network’s global complexity and vice versa. Each approach complements the other, as they capture information from different perspectives. Fractal analysis has been used to investigate retinal vascular development in many circumstances. The box-counting fractal dimension (D_B_) method is the most widely utilized to analyze retinal vasculature complexity [53,60,68], thus being selected for our protocol. Box-counting fractal dimension quantifies how complex and space-filling a branching pattern is by counting how many grid boxes contain vessel segments as the box size decreases. In the context of the retinal vasculature, this measure reflects the geometric complexity and curvature of the vessel network.

Although D_B_ is a powerful tool, it can be nonspecific, as different patterns with similar D_B_ can remain qualitatively very different [71]. To mitigate this, lacunarity used in conjunction with fractal dimension characterizes the heterogeneity and symmetry of gaps surrounding an object and together form a more specific impression of the vascular network [71]. Lacunarity measures how evenly or unevenly a pattern fills space, capturing the size and variability of gaps within a structure. Low lacunarity indicates a uniform, tightly packed network with consistent spacing between elements, whereas high lacunarity reflects larger, more irregular gaps and a less cohesive pattern.

In this publication, we report the development of a standardized computational approach that captures and measures structural and pathological features in a mouse retina flat mount in a rapid and reproducible fashion in ImageJ tables, using the software and plugins in Table 3. More specifically, it quantifies the total retina size (μm^2^), the percent vascularized area (%), and the proportion of area of significant vascular disorganization (*meshes*) that have no definable network structure (%). Then, in chosen regions of interest at the periphery and midperiphery, we calculate VD (ratio), BL (μm), BT (μm), BN, JN, TP, number of cell clusters (CCs, microaneurysm-like structures), D_B_ (unitless), and lacunarity (Λ̅ unitless).

## Equipment

1. Laptop or desktop computer with the required software installed. We used a MacBook Pro (2.4 GHz Quad-Core Intel Core i5/ 8 GB 2133 MHz LPDDR3/macOS 15.7.4 24G517).

Minimum requirements to download the latest versions of FIJI are:

a. Windows 10 or later, x86–64 or arm64

b. macOS 11 “Big Sur” or later, Apple Silicon or Intel

c. Ubuntu 22.04 LTS or later, x86-64 or arm64

d. Any other system with a Java 21 runtime, except plugins using native libraries (e.g., 3D Viewer)

## Software and datasets

**Table 3.** Software and plugins used in the FIJI retinal vasculature analysis protocol

Type	Resource	Version	Date	License	Access (free or paid)
Software	FIJI	2.16.0/1.54p	October 15, 2024	GNU General Public License	Free
Plugin 1	Analyze skeleton	https://imagej.net/plugins/analyze-skeleton/index			Free
Plugin 2	Skeletonize (2D/3D)	https://imagej.net/plugins/skeletonize3d			Free
Plugin 3	Unsharp mask (USM)	https://imagej.net/ij/docs/guide/146-29.html#toc-Subsection-29.11			Free
Plugin 4	Median filter	https://imagej.net/ij/docs/guide/146-29.html#toc-Subsection-29.11			Free
Plugin 5	Subtract	https://imagej.net/ij/docs/guide/146-29.html#toc-Subsection-29.9			Free
Plugin 6	Contrast-limited adaptive histogram equalization (CLAHE)	https://imagej.net/imagej-wiki-static/Enhance_Local_Contrast_(CLAHE)			Free
Plugin 7	MorphoLibJ	https://imagej.net/plugins/morpholibj			Free
Plugin 8	BoneJ	https://imagej.net/plugins/bonej https://bonej.org			Free
Plugin 9	FracLac	https://imagej.nih.gov/ij/plugins/fraclac/FLHelp/Introduction.htm			Free
Plugin 10	Non-local means denoise (NLM)	https://imagej.net/plugins/non-local-means-denoise			Free
Plugin 11	Minimum Length Prune	https://imagej.net/plugins/analyze-skeleton/index			Free

## Procedure

**A. Set the scale to convert pixels to μm**

1. *Analyze* > *Set Scale*…

2. Use the line tool to draw over the scale bar.

3. Put in the known distance from the scale on your image and the units of measurement.

**B. Set your measurements**

1. *Analyze* > *Set Measurements*…

2. Select *Area, Display label, Area fraction*, and *Add to overlay*.

**C. Outline the total retinal area**

**Figure 1.**
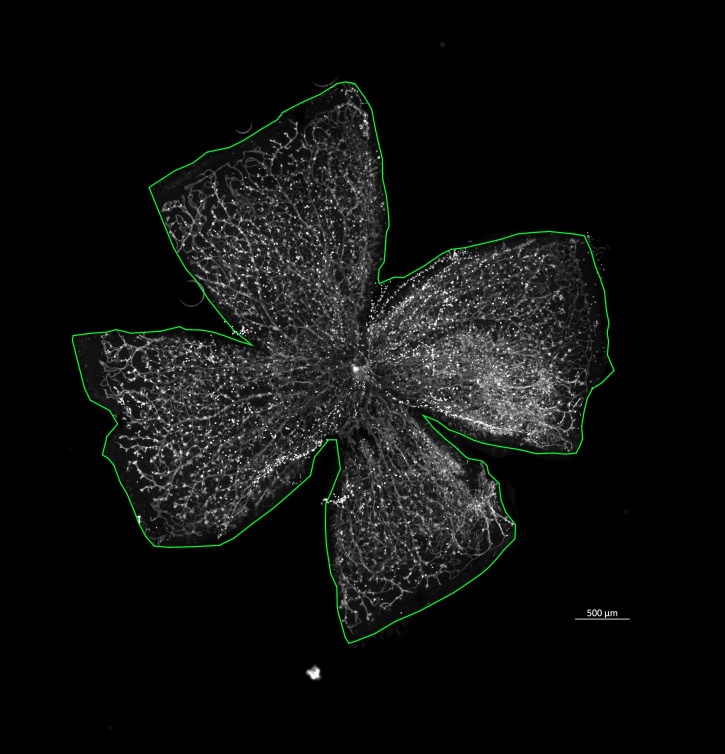
Whole retina flat mount image of the FZD4 mouse model with the outer border of the retina outlined. Scale bar, 500 μm.

1. Select the polygon selection tool.

2. Outline the outer borders of the retina (Figure 1).

3. Add the overlay (*Image > Overlay > Add Selection*) to the ROI manager (*Image > Overlay > To ROI manager*).

4. Measure the outline (*Analyze > Measure*) and record the value.

**D. Total retinal vascularized area**

**Figure 2.**
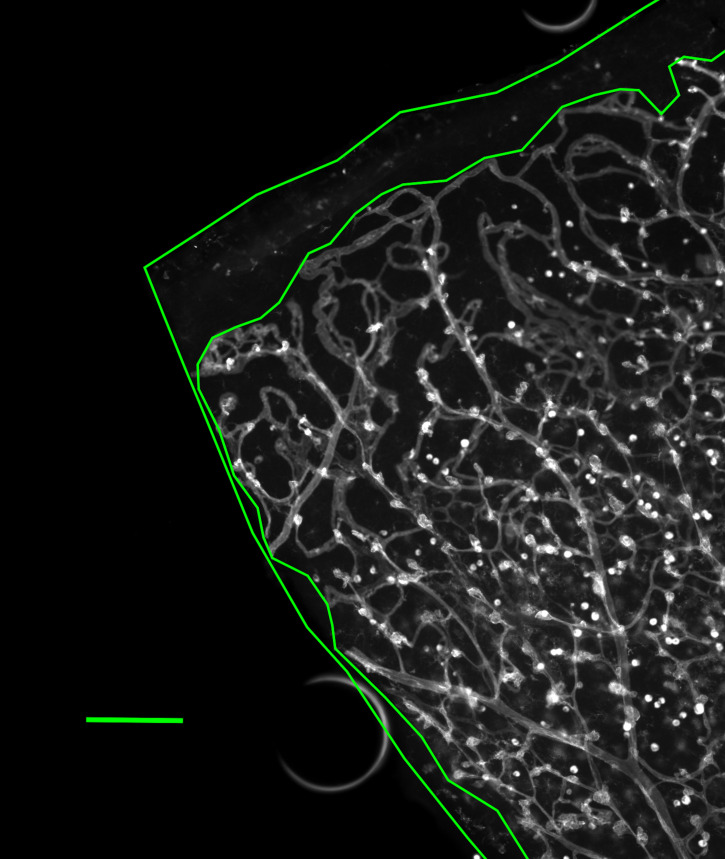
Enlarged corner of the whole retina image, highlighting the outer border of the retina and the peripheral border of the vascularized area. Scale bar, 200 μm.

1. Select the polygon selection tool.

2. Outline the vascularized area of the retina up to the periphery (Figure 2).

3. Add as an overlay (*Image > Overlay > Add Selection*) to the ROI manager (*Image > Overlay > To ROI manager*).

4. Ensure that you have the vasculature outline selected when you are measuring. Measure the outline (*Analyze > Measure*) and record the value.

**E. Saving representative ROIs from the periphery and midperiphery**

**Figure 3.**
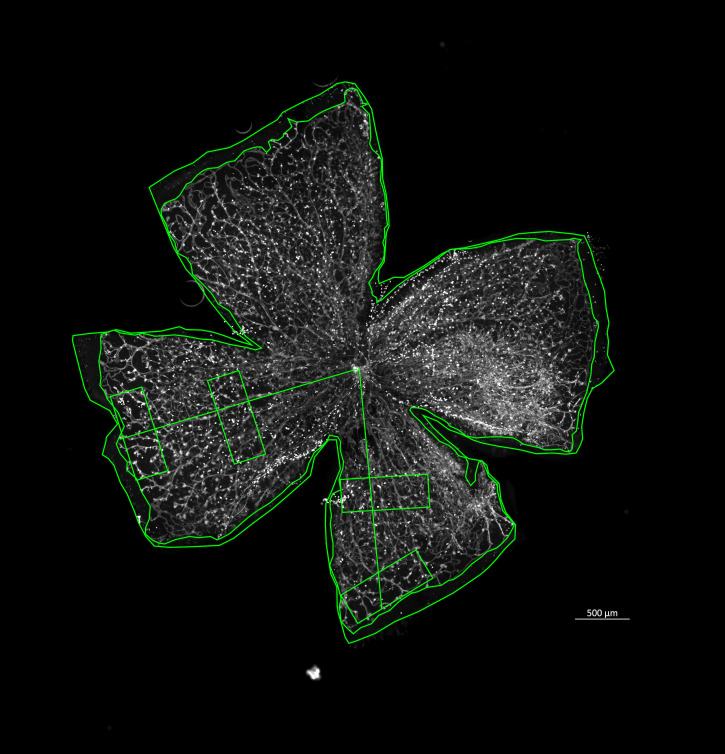
Whole retina flat mount image of the FZD4 mouse model with representative 300 × 800 μm regions of interest (ROIs) at the periphery and mid-periphery selected in the right and left lower quadrants of the retina, which do not contain meshes. The outer border of the retina and the vascularized area are also outlined. Scale bar, 500 μm.

1. Choose a quadrant of interest.

2. Use the *Rotated Rectangle* tool to draw a 300 × 800 μm (depth × width) rectangle at the periphery of the retina following the angle of vessels (Figure 3). Avoid analyzing areas that are not representative of the overall structure of the retinal vasculature.

3. Add the ROI as an overlay to the ROI manager.

4. Draw a second 300 × 800 μm rectangle overlay at the mid-periphery of the retina in the same quadrant and add it to the ROI manager (Figure 3).

*Notes: The line tool can be used to precisely locate the midway point from the center and periphery, which is what the mid-periphery ROI should cover. This is done by drawing a straight line from the center to the periphery, adding it as an overlay, and noting where the overlay numerical label marker appears, as this is the exact midpoint of that straight line.*

5. Prioritize getting a quantifiable representative area, i.e., the mesh may preclude measurements in a specific region, hence the need to capture the mesh separately.

6. Repeat steps E2–5 for all quadrants of interest.

7. Duplicate each overlay from the ROI manager in sequential order to produce individual images of the selected peripheral and mid-peripheral regions in each quadrant of the retina.

8. Save images for processing.

**F. Processing ROI images**

**Figure 4.**
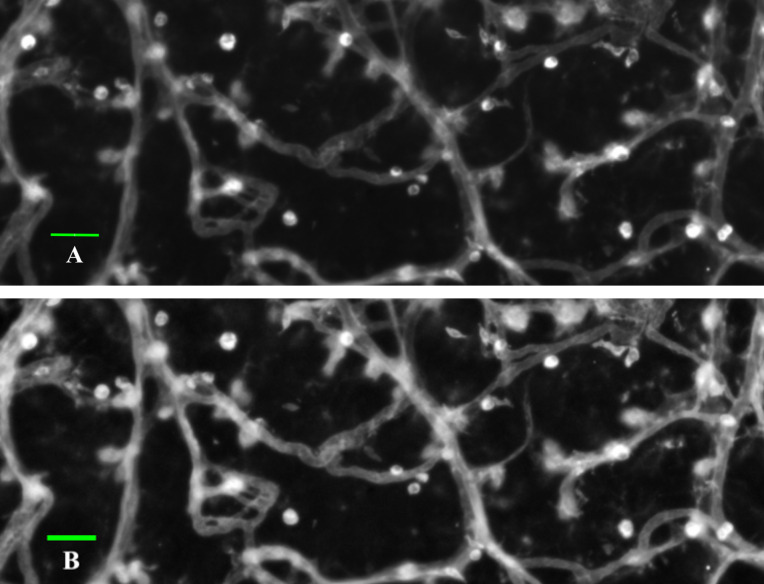
Representative images before (A) and after (B) contrast-limited adaptive histogram equalization (CLAHE) filtering. Scale bar, 50 µm.

1. Open the previously saved ROI image and duplicate it.

2. Convert the image to grayscale 8-bit if the image is in color (*Image > Type > 8-bit*).

3. Enhance contrast to better outline the vessels using the *Enhance Local Contrast* feature [*Process > Enhance Local Contrast (CLAHE)*] (Figure 4).

*Notes:*

*1. This can enhance noise.*

*2. Blocksize is the area that is equalized.*

*3. Histogram bins are the number of bins used in the histogram; use the maximum number of pixels in the block up to the number of grayscale values (256 for an 8-bit image).*

*4. The maximum slope is the amount of contrast enhancing. Slope = 1 is the original image.*

**Figure 5.**
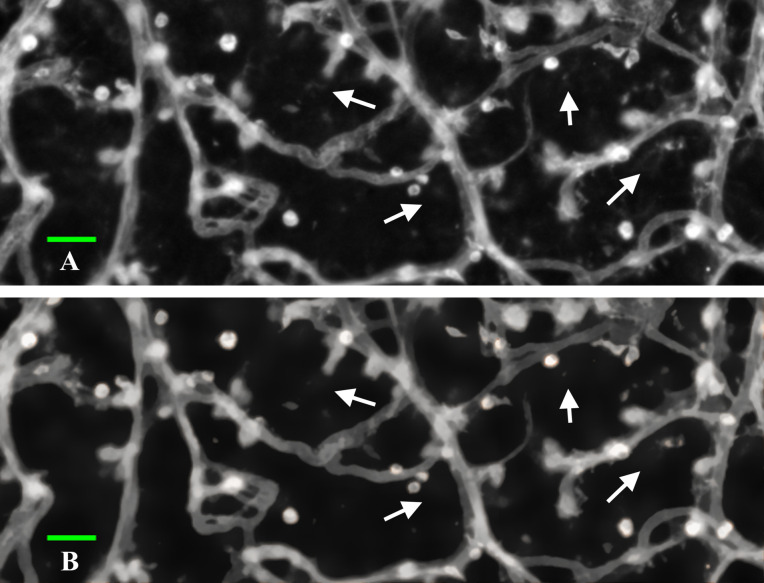
Representative before (A) and after (B) images after using non-local means (NLM) filtering. White arrows point to general areas where NLM successfully reduced noisy, heterogeneous sections of the image. Scale bar, 50 µm.

4. Filter any Gaussian background noise using the non-local means denoising plugin (*Plugins >Non-local means denoising*) (Figure 5).

*Notes:*

*1. This can eliminate details such as small vessels.*

*2. Sigma is the range or variation in noise. In practice, it is similar to the degree of averaging of the signal by the plugin.*

*3. Smoothing factor is the level of smoothing. Typically, keep it at 1.*

*4. Auto-estimate when you can, but do it manually if the level of filtering is too high or low.*

**Figure 6.**
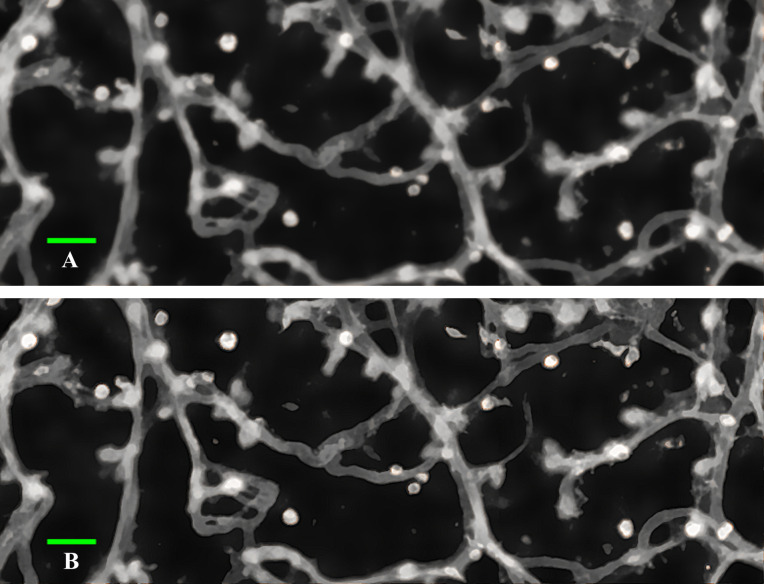
Representative before (A) and after (B) images after using Unsharp Mask filtering. Scale bar, 50 µm.

5. Use the *Unsharp Mask* filter (*Process > Filters > Unsharp Mask*) to enhance the edges of the vessels. This helps maintain the distinction between vessels and the details of the structure (Figure 6).

*Notes:*

*1. This can enhance noise and worsen the heterogeneous staining of vessels, which can create artifacts when binarizing.*

*2. The radius is the standard deviation of the Gaussian blur that is subtracted to enhance edges.*

*3. Mask weight is the strength of filtering, where 1 represents infinite weight. Preview the image to optimize the parameters before finalizing.*

**Figure 7.**
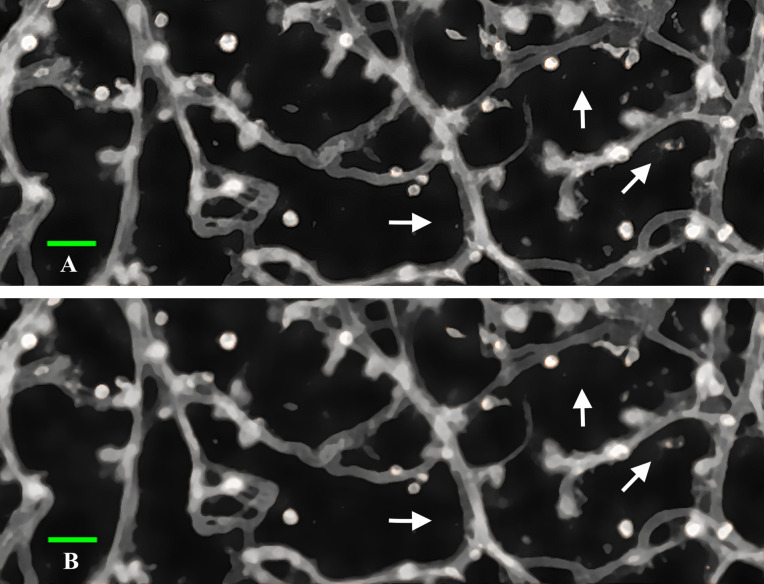
Representative before (A) and after (B) images after using Median filtering. White arrows indicate areas where high-intensity noise around edges was smoothed. Scale bar, 50 µm.

6. Use the median filter (*Process > Filters > Median*) to remove salt-and-pepper noise, i.e., high and low intensity specs in the background (Figure 7).

*Notes:*

*1. This should preserve edges better than Gaussian, mean, or other basic local filters, but it will do some smoothing.*

*2. Radius is the radius of the pixels compared to set the median; the higher value, the more smoothing. Optimize by using previewing.*

7. Use the *Subtract* function (*Process > Math > Subtract*) to reduce the values of all the pixels in the image by a set amount chosen by you. This can be used as the last attempt in removing a loud patch of noise that other filtering functions could not delete.

*Note: This is a brute-force way to remove any remaining background noise. Subtract by the value of the pixels in the darkest part of the background or the parts you want to eliminate.*

**Figure 8.**
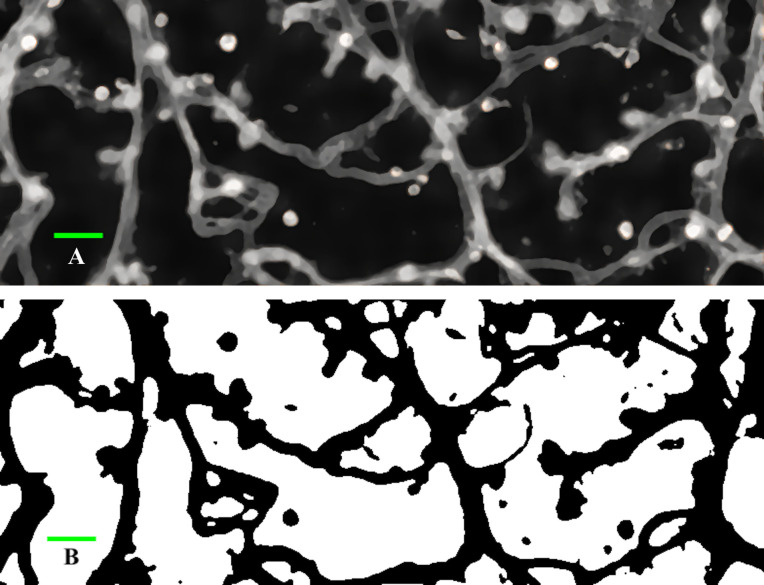
Representative image of a region of interest (ROI) before (A) and after (B) it has been converted to a binary image. Scale bar, 50 µm.

8. Binarize the image by first setting the threshold for LUT (*Image > Adjust > Threshold*). Adjust the threshold so that the black and white image represents the original.

9. Convert the new image to a binary image (*Process > Binary > Convert to Mask*) (Figure 8).

*Note: Convert the image pixel values to 255 (if 8-bit) for foreground and 0 for background.*

**Figure 9.**
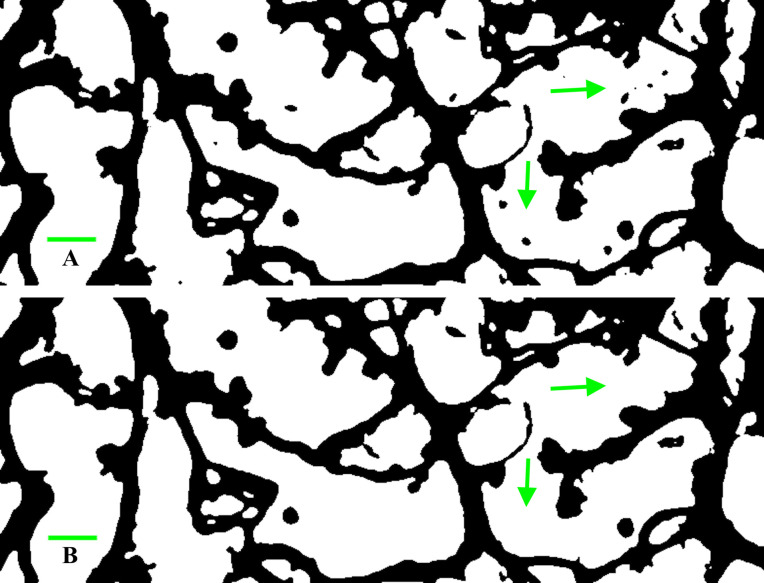
Representative image before (A) and after (B) the filtering process, removing objects smaller than 100 µm^2^. Arrows point to some areas that were cleared. Scale bar, 50 µm.

10. Analyze particles (*Analyze > Analyze Particles…> size=100-Infinity, Show = Masks*) to filter out particles with an area less than 100 μm^2^ (Figure 9). This size was chosen to avoid filtering out real and large structures in the image. The remaining unconnected CCs do not end up having a significant impact on the results.

a. Outline all structures and proceed with whatever operation you have set, such as counting, measuring, or removing.

b. Size sets the range of area you intend to consider.

c. Show = Masks tells the program to show the structures that fit within your range, effectively filtering out everything below or above it. In this case, as the upper bound is “infinity,” it filters any object less than 100 μm^2^.

11. Use morphological filters (*Plugins > MorphoLibJ > Morphological Filters*) to further filter the image to maintain the overall structure of the vasculature.

*Note: This is useful for smoothening the borders of the vascular structure and dealing with holes, but it can significantly impact the morphology when holes and branches are changed or eliminated.*

**Figure 10.**
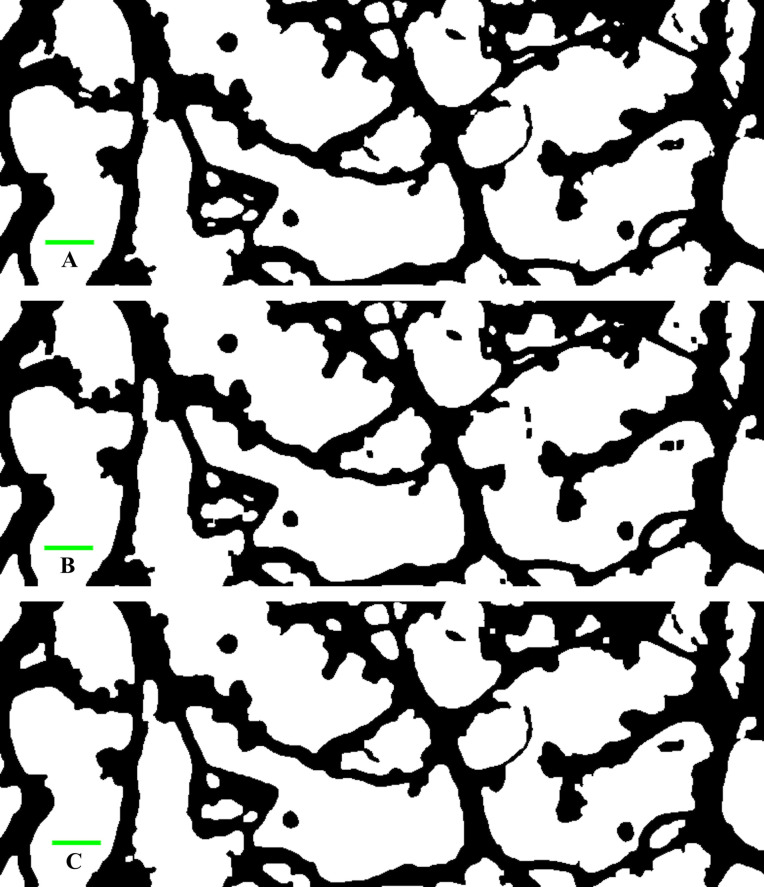
Examples of the effect of morphological filters from the original image (A) and after an opening filter (B) or closing filter (C). Scale bars, 50 µm.

a. Opening: Eroding and then dilating the binary image by a set number of pixels using an “element.” In other words, the shape and size of the brush that will scrub over the image. This is useful for smoothening shape borders, pruning thin unwanted branches, or widening gaps.

b. Closing: Dilating then eroding using a set element. Useful for filling holes and gaps and smoothing borders.

c. Filters can be used in combination/series to great effect (Figure 10).

i. Opening and closing operation with a square shape and pixel size of 1–2 is typically used.

ii. Directional morphological filters may be better at highlighting thin structures, since the elements are lines rather than blocks.

**G. Measure vessel density, lacunarity, and tortuosity**

1. Based on the binary image, calculate the vessel density, Db, and Lac.

2. *Plugins > BoneJ > Fraction > Area/volume fraction.*

3. *Plugins > MorphoLibJ > Analyze > Average Thickness.*

4. Fractal analysis

a. *Plugins > Fractal Analysis > FracLac*. In the window, click the BC box for box-counting dimension, set the settings, click *Ok*, then click *Scan*.

b. Settings: Default except for: Lock white background, max grid size of 30 pixels, and regression graphic option checked.

c. Take the average Db (D =  ∑D/ ∀ ∈  for  D) and the lacunarity (L) = ∑Λ/ ∀ ∈

**H. Skeletonization**

**Figure 11.**
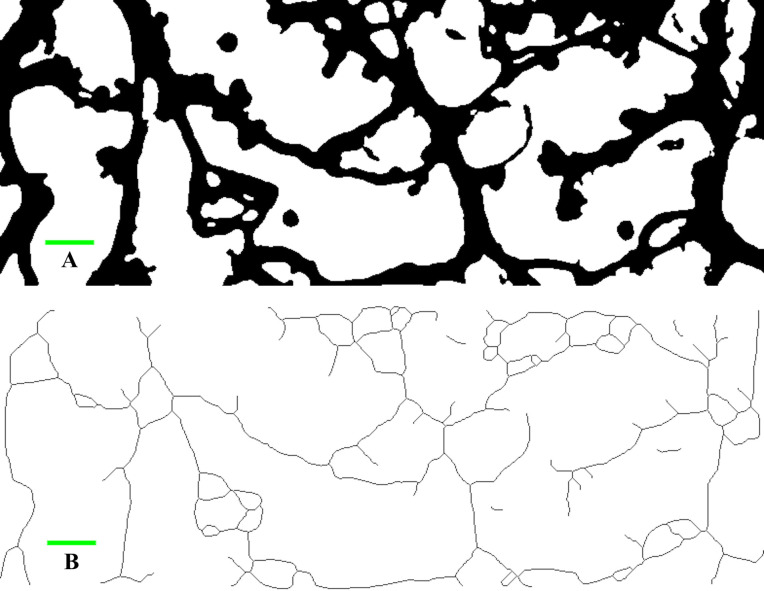
Representative image demonstrating the effect before (A) and after (B) skeletonization. Scale bar, 50 µm.

1. Collapsing down the structure in the binary image so that all that is left is the connected centerlines of all the structures (Figure 11).

2. *Plugins > Skeleton > Skeletonize (2D/3D)*

**I. Measure branch length and number of branch points**

1. Based on the skeleton, measure the average branch length and number of branches, junctions, and triple points.

2. *Plugins > BoneJ > Analyze Skeleton* (no pruning, nothing selected).

**J. CC counting**

**Figure 12.**
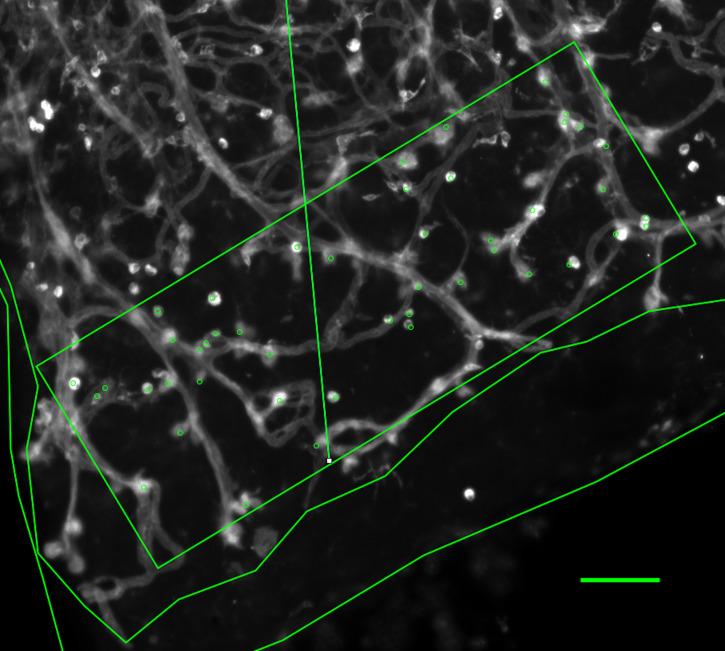
Cell clusters (CCs) are marked for counting in a region of interest (ROI). Scale bar, 100 µm.

1. Using the multi-point selection tool, mark and count each CC in the ROI images. CCs are roughly spherical bulbs of cells budding off vessels (Figure 12).

2. Measure the overlay and take the total number of point entries.

**K. Grading areas of vascular disorganization**

**Figure 13.**
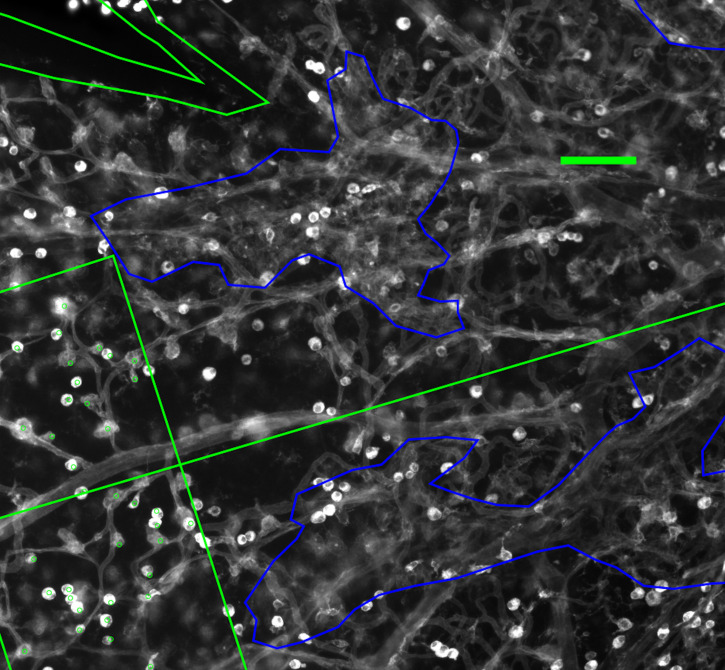
Example Grade 1 mesh outlined in blue. Grade 1 is an area with significant noise where vessels appear to be fused together but there is still some ordered structure. Scale bar, 100 µm.

**Figure 14.**
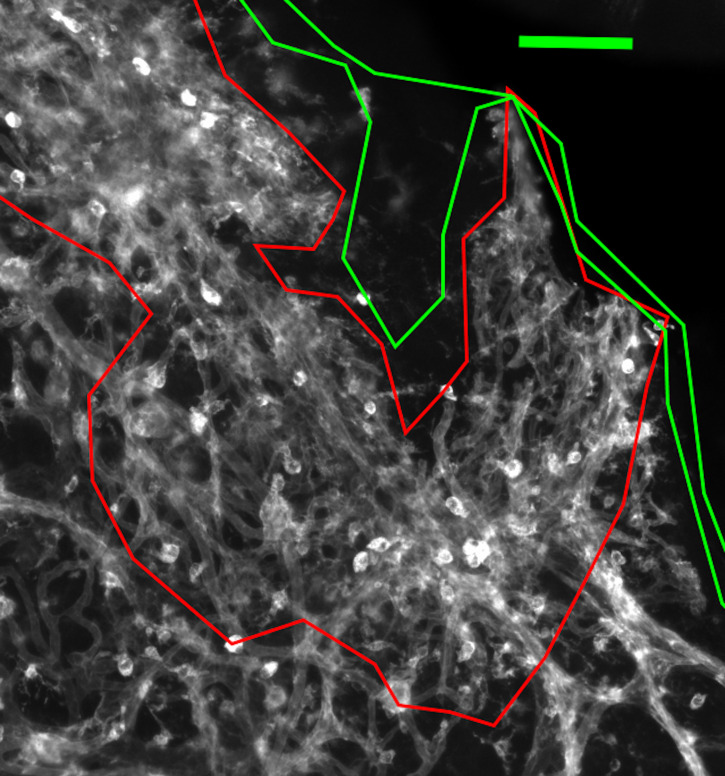
Example Grade 2 mesh outlined in red. Grade 2 is an area with significant noise that is an almost completely disordered mess of endothelial cells. The occasional defined vessel is not enough to classify it as Grade 1. Scale bar, 100 µm.

1. Open the original retina image.

2. Outline the areas of vasculature disorganization using the *polygon selection* tool and measure the area similar to the methods of the total vascularized area noted above (Figures 13 and 14).

**L. Summary**

**Figure 15.**
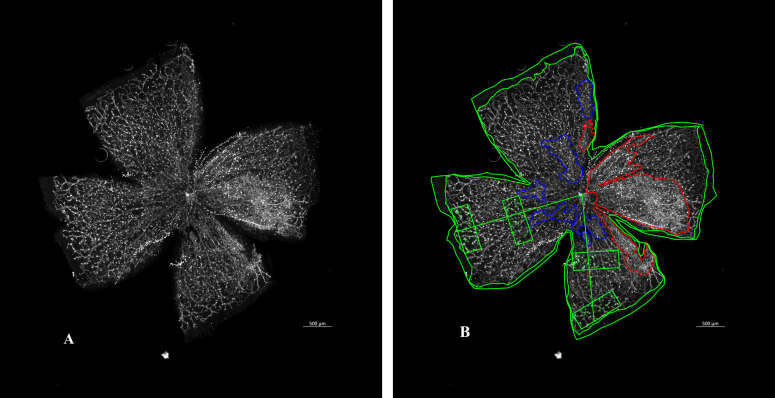
Whole retina flat mount image of the FZD4 mouse model. (A) Before and (B) after total retina and vascularized area, Grade 1 and 2 meshes (blue and red, respectively), periphery and mid-periphery 300 × 800 µm regions of interest (ROIs), and cell clusters (CCs) are outlined and labeled. Scale bar, 500 µm.

**Figure 16.**
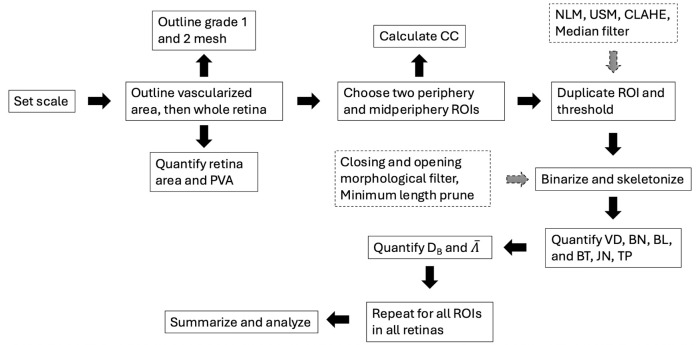
Workflow diagram for the digital image analysis of FZD4 models and wild-type (WT) controls. Abbreviations: NLM, non-local means; USM, unsharp mask; CLAHE, contrast-limited adaptive histogram equalization; CC, cell cluster count; VD, vessel density; BN, number of branches; BT, branch thickness; BL, branch length; JN, number of junctions; TP, number of triple points; Λ̅, lacunarity; D_B_, box-counting fractal dimension; ROI, region of interest.

1. At the end of the analysis, you will have images of the whole retina flat mount (Figure 15), the different ROIs, the binary ROIs, the skeleton ROIs, and the FracLac outputs.

2. Measurement generated by the workflow (Figure 16):

a. Retinal area (μm^2^) and vascularized area/fraction (ratio).

b. Percent areas of Grade 1 and 2 meshes (%).

c. CC count of ROIs (count).

d. Vessel density (ratio).

e. Vessel average branch length and diameter (μm).

f. Number of junctions and triple points (count).

g. Db and lacunarity (unitless).

## Validation of protocol

This protocol has been used and validated in the following research article:

- Feridooni et al. [72] Sphingosine-1-phosphate receptor 2 inhibition ameliorates familial exudative vitreoretinopathy models. *Journal of Biological Chemistry* (Figures 2–5).

## General notes and troubleshooting

**General notes**

1. Not all tools should be used all the time, and each image requires different degrees of enhancement, filtering, etc. The goal is to get an accurate representation of the vascular structure; thus, we recommend the application of our protocol according to your best judgment for the highest quality data for your purposes. For this reason, we do not prescribe specific settings for each parameter as the image is processed.

2. Always save after every major step. There is no reliable undo or redo button in FIJI. It is important to save the final versions of selections, binary, and skeleton images. ImageJ guides for basic functions are available at https://imagej.nih.gov/ij/docs/guide/ and https://imagej.net (New ImageJ Wiki).

3. For minimum length prune, take the script listed on the Analyze Skeleton page and save it as a plugin. Instructions on how to do so are here available at https://imagej.net/scripting/.

4. Batch analysis in FIJI and FracLac can be incorporated depending on the level of automation (macros) you implement.

5. You can easily create your own macro by using the Macro Recorder tool or scripting to automate any steps to save time (https://imagej.net/scripting/macro).

6. Retinal vasculature labeling, image acquisition, and quality would evidently impact results. Our full method is presented in our validation study [73], and including this methodology is beyond the scope of this paper, as it is focused on computational analysis. To briefly summarize, retinal whole mounts were stained with Alexa Fluor 594–conjugated GS-IB4 lectin to visualize the vascular endothelium, as previously described [73–77]. Images were acquired at 10× magnification using a Zeiss Axio Imager Z2 microscope equipped with a motorized digital stage and MosaiX software in Axiovision 4.8, with all samples imaged under identical acquisition settings.

**Troubleshooting**

**Problem 1**: Unexpected error.

Possible cause: Using old versions of plugins, or an aspect of the underlying code is causing an error.

Solutions: Refer to https://imagej.net/plugins/updater for more details on how to regularly update all plugins and FIJI. Get in contact with the broader image analysis and FIJI development community on https://forum.image.sc for custom solutions and direct contact with developers.
